# Frontal and Parietal Activities Associated With Different Inhibitory Processes in a Stroop‐Matching/Stop‐Signal Task: A Channel‐Wise fNIRS Study

**DOI:** 10.1111/psyp.70098

**Published:** 2025-07-07

**Authors:** Armando dos Santos Afonso Junior, Walter Machado‐Pinheiro, Luiz Renato Rodrigues Carreiro

**Affiliations:** ^1^ Mackenzie Presbyterian University Sao Paulo Brazil; ^2^ Fluminense Federal University Rio das Ostras Brazil

## Abstract

Inhibition is an important component of cognitive control that encompasses multiple processes, such as interference control, inhibition of prepotent responses and suppression of ongoing responses. Frontal and temporoparietal regions of the cortex are implicated differently in inhibitory functions. The Stroop‐matching/stop‐signal task is a recent task that uses Stroop stimuli and stop‐signals to create conditions that allow the investigation of the three forms of inhibition aforementioned. The task provides a way to distinguish the effect of these inhibitions as well as their interactions using a single task. The present study used functional near‐infrared spectroscopy (fNIRS) to assess frontal and temporoparietal activations during the Stroop‐matching/stop‐signal task. The main objective was to investigate which cortical regions each inhibitory function would recruit during this task. Fifty‐two young adults (mean age = 21.4, SD = 3.44) participated. Performance results indicated the effects previously found in the Stroop‐matching/stop‐signal task. fNIRS results showed that the left inferior frontal cortex (IFC) and the bilateral intraparietal sulcus are involved in interference control; the left IFC also showed activation in inhibition of prepotent responses; and the right IFC was involved in the suppression of ongoing responses. The interaction between suppression of responses and the other two forms of inhibition lead to deactivation of frontal and parietal areas. Thus, each form of inhibition demanded by the Stroop‐matching/stop‐signal task seems to recruit specific cortical regions, supporting the distinction between inhibitory components at the neurophysiological level.

## Abstract 2

This study revealed different patterns of brain activity associated with three inhibitory components (interference control, inhibition of prepotent responses and suppression of ongoing responses). Inhibition of prepotent and ongoing responses seems to rely more on frontal regions, whereas interference control also recruits parietal regions to deal with distractive information. Moreover, when suppression of ongoing responses interacts with other inhibitory processes, it leads to a deactivation of inhibition‐related brain regions. The Stroop‐matching/stop‐signal task was able to distinguish different inhibitory effects using a single task.

## 1 Introduction

### 1.1 Inhibitory Processes as a Component of Cognitive Control

Cognitive control^1^ is a regulatory process that optimizes deliberate goal‐directed behaviors while resisting automaticity. Psychometric and neuropsychological evidence shows that cognitive control is composed of united and diverse processes—they share some behavioral and neural characteristics, but they can still be fractionated (Friedman and Robbins 2022). Even though some cognitive control components are already well accepted, such as working memory, cognitive flexibility, and inhibition (Diamond 2013; Miyake et al. 2000; Zink et al. 2021), further examination of each construct can lead to a better understanding of their contribution to psychiatric conditions and neural dysfunctions (Friedman and Robbins 2022). This paper focuses on inhibition as a core component of cognitive control, even being considered the common factor to every cognitive control function (McKenna et al. 2017; Miyake et al. 2000; Miyake and Friedman 2012).

Inhibition is a multifaceted concept sometimes used to refer to a set of cognitive processes that allow the effortful control of behavior through the suppression of interfering stimuli or actions (Nigg 2017). Influential models of cognitive control place inhibition as an essential component of organized behavior, whereas disinhibition can lead to several problems, such as academic underachievement (Gerber et al. 2021), road accidents (Walshe et al. 2017), suicidal risks (Sandoval et al. 2022) and are part of psychiatric disorders characterized by impulsive actions (Bari and Robbins 2013; Lipszyc and Schachar 2010), including Tourette syndrome (Wylie et al. 2013) and ADHD (Pani et al. 2013). Multiple inhibitory components were already elucidated in the literature (Morra et al. 2018). Within the profusion of proposed taxonomies, a straightforward approach to differentiate inhibitory processes involves distinguishing between the inhibition of motor responses and interference (Wostmann et al. 2013). Interference control acts by suppressing distractive information that causes conflict and hampers behavior. In its turn, inhibition of motor responses involves the inhibition of irrelevant or unnecessary actions. Response inhibition can be further divided according to the stage of processing in which the response is stopped (Gillespie et al. 2022). Inhibition of prepotent responses acts by inhibiting responses that are automatic and fast before they are initiated, that is, restraining the emission of response. Conversely, the suppression of ongoing responses interrupts an action that has already been initiated and is running to completion (Raud et al. 2020).

### 1.2 Neural Bases of Inhibitory Processes

Cognitive control is mediated by the prefrontal cortex (PFC). Even though the different frontal lobe regions and other networks spread throughout the brain have been shown to be involved in different aspects of cognitive control, the PFC is highly praised for its role in the coordination of cognition and behaviors in accordance with internal goals (Friedman and Robbins 2022; Menon and D'Esposito 2022). The dorsolateral prefrontal cortex (DLPFC) is the part of the PFC that is viewed as a major hub of top‐down control over other lower‐order frontal regions (Badre and Nee 2018). The DLPFC is a portion of the mid‐lateral prefrontal cortex that comprises at least two regions with different cytoarchitectures, Broadmann areas 9, on the superior frontal gyrus and middle frontal gyrus and 46, on the middle frontal gyrus (Petrides 2005). Several brain regions are connected to the DLPFC, including the thalamus, basal ganglia, hippocampus and parietal and posterior temporal areas (Jung et al. 2022) which may explain its involvement in a variety of cognitive functions, such as working memory, planning, reasoning and inhibition (Miller and Cummings 2007). Experimental and clinical studies showed increased DLPFC activity in tasks that requires distractor suppression (Colombo et al. 2015; Floden and Stuss 2006) and inhibition of motor responses (Miyake et al. 2000; Oldrati et al. 2016) which support its putative role in different forms of inhibition. An influential framework for the role of the DLPFC in inhibitory control is the conflict‐monitoring theory that attributes to the anterior cingulate cortex the function of monitoring interference and alerting the DLPFC in case of uncertainty, stimulus or response conflict. After receiving the alerting signals, the DLPFC can exert top‐down control over other systems involved in the resolution of the task (Banich 2019).

The inferior frontal cortex (IFC, also known as ventrolateral prefrontal cortex or cited in inhibitory studies as inferior frontal gyrus) comprises Broadmann Areas 44 (pars opercularis), 45 (pars triangularis) and 47/12 (pars orbitalis) (Petrides and Pandya 2002) and is commonly pointed out as an important region to the inhibition of motor responses (Suda et al. 2020). The IFC role in interrupting actions that have already been initiated is well accepted and has been supported by experimental (Aron et al. 2014; Chen et al. 2020; Suda et al. 2020) as well as lesion studies (Choo et al. 2022). The right IFC is directly connected to the subthalamic nucleus via a monosynaptic pathway forming an inhibitory fronto‐basal‐ganglia network (Chen et al. 2020). Interruption of initiated responses occurs when the subthalamic nucleus (STN), upon receiving IFC signals, communicates with the substantia nigra in order to inhibit thalamus activity (Aron et al. 2014). The presupplementary motor area (preSMA) also receives top‐down signals from the IFC to perform motor inhibitions (Schaum et al. 2021). One hypothesis is that the IFC may trigger the STN via the preSMA, although this is still unclear and they may constitute separated pathways originated in the IFC and that interrupts actions (Aron et al. 2014). Although the right IFC is consistently associated with interrupting initiated actions, the left IFC seems to be involved in the inhibition of prepotent responses (Kramer et al. 2013; Swick and Chatham 2014). That is, the right IFC cancels actions and the left IFC restraint actions (Kramer et al. 2013), which provides neurofunctional basis to the diversity of response inhibitions. Importantly, the IFC is connected to the DLPFC (Jung et al. 2022), but their joint role in inhibition is not clear.

Even though frontal activity has been historically conceived as the main source of cognitive control, a range of studies has shown that other regions also contribute, most importantly the parietal lobe (e.g., Kolodny et al. 2017; Nee 2021). Anatomical findings showed that the posterior parietal cortex (PPC) in particular has reciprocal projections with PFC regions associated with inhibitory functions (Fox and Raichle 2007). In fact, the fronto‐parietal network, also known as the central executive network, includes the DLPFC and the posterior parietal cortex (Friedman and Robbins 2022; Menon and D'Esposito 2022), and acts as a flexible hub for the coordination of cognitive control supposedly by modulating other brain networks (Marek and Dosenbach 2018). The intraparietal sulcus (IPS), part of the posterior parietal cortex, seems to be closely related to frontal functions including cognitive control, which indeed is shown by studies that show that the IPS integrates motor, sensory, and cognitive information that are task relevant while being mediated by the DLPFC (Gottlieb 2007). Moreover, the IPS shows an increase in activity when target stimuli appear among distractors (Chun and Marois 2022) and has been associated with tasks that require interference control (Zysset et al. 2001). It is also activated in the inhibition of prepotent responses (Chikazoe et al. 2009; Kolodny et al. 2017) and suppression of initiated actions (Osada et al. 2019). IPS can therefore be implicated in the inhibition of perceptual information and motor commands (Kolodny et al. 2017). Close to the IPS, the TPJ is another region that has received more attention in the inhibition of prepotent responses (Ghin et al. 2022; Kolodny et al. 2017; Osada et al. 2019) and may be crucial to behavioral control. Therefore, other regions, posterior to the PFC, seem to be involved in different forms of inhibition, even if they are not part of the inhibition debate as much as frontal regions.

### 1.3 Measures of Inhibitory Processes

Paradigms that tap interference control include tasks such as Simon and Eriksen flanker task, where distractive stimuli activate different potential responses (Mirabella 2022). On the other hand, response inhibition paradigms include the go/no‐go task for inhibition of prepotent responses and the stop‐signal task for suppression of ongoing responses (Raud et al. 2020). The main difference between these two tasks is that the go/no‐go task requires the restraint of a potential “go response” that was previously established as automatic by manipulating the frequency of “no‐go trials” whereas the stop‐signal task requires the inhibition of an ongoing response in face of a cue that is presented after response was initiated.

One distinctive cognitive paradigm in the inhibition literature is the Stroop task. In the traditional version of the Stroop task, participants are asked to read color‐words (e.g., the word “red”) or name the color of colored bars (e.g., a red bar). The typical finding is that participants are slower or make more errors in incongruent conditions, in which they have to read words whose colors are different than their meaning (e.g., the word “red” colored in green), in comparison to conditions in which they have to read color‐words in neutral color (e.g., the word “red” colored in black) or to name colored bars (Scarpina and Tagini 2017; Stroop 1935). The Stroop task is a hallmark in the study of cognitive control, and it has been used for almost a century, but the nature of its conflicts is still discussed (Banich 2019). Some authors argue that the Stroop task involves a conflict between competing responses (i.e., color naming and word reading) while others argue that the conflict happens prior to the response level, at the phonological or semantic levels (Shichel and Tzelgov 2017). This is still a topic of debate (Machado‐Pinheiro et al. 2024). Similarly, it is not clear if the Stroop task demands interference control mechanisms to handle interference caused by the distractive word of the Stroop stimulus or if it demands inhibition of the prepotent response, since participants must inhibit the automatic response of reading the word to complete the task successfully (Stahl et al. 2014).

The Stroop‐matching task is an alternative version of the classic task that does not share the same uncertainty issues regarding its conflicts. In a Stroop‐matching task, a probe is presented with a Stroop stimulus (i.e., a colored color‐word). The probe is a stimulus formed by a single attribute of the Stroop stimulus, either color or word. It usually is a color‐word in neutral ink (e.g., the word “red” colored in black) or a colored nonword stimulus (e.g., a red bar). The participants are instructed to compare one attribute of the Stroop stimulus (color or word) with the probe while ignoring the other Stroop attribute (Dyer 1973; Treisman and Fearnley 1969). Interference effects are observed when there is a mismatch between the Stroop stimulus and the probe. Importantly, conflict arises only if the comparison made between Stroop stimulus and probe involves different types of attributes (color‐word or word‐color) but not if it is between the same attribute (color–color or word‐word). That is, interference effects can only be observed when participants match the color of the Stroop stimulus with a neutral colored word or when they match the Stroop word with a colored bar (between‐attributes matches). Trying to inhibit the automatic reading of the Stroop word is not necessary for conflict in this task and even when the Stroop color is a distractive attribute, performance can be impaired (Dittrich and Stahl 2016; Machado‐Pinheiro et al. 2010; Treisman and Fearnley 1969). This effect is explained in terms of the need to use distinctive cognitive modules to process colors and words. When the comparison is made between target attributes of the same type (e.g., colors) and the distractor attribute is from a different type (e.g., word) then only the module responsible to process the relevant attribute needs to be activated and thus the distractor attribute is not processed and does not cause interference. However, when the task requires the processing of colors and words then both modules are activated and the distractors interfere with the response (Dittrich and Stahl 2016).

A specific version of the Stroop‐matching task presents the Stroop stimulus alongside two colored bars as probes and asks the participants to compare the Stroop word with the two bars in order to choose the bar whose color matches the meaning of the Stroop word (Afonso Jr et al. 2020; Machado‐Pinheiro et al. 2024). One colored bar is the right response option and matches the Stroop word while the other colored bar is a wrong option that may be the same as the Stroop color or not. Therefore, the word is the target attribute of the Stroop stimulus and its color is a distractor. In this design, two sources of conflicts are observed. The first conflict arises in the incongruent condition when the two attributes of the Stroop stimulus are different and it is called Congruency Effect. Notably, the Congruency Effect reflects the interference caused by the Stroop color, which is a distractor in this task but does not elicit an automatic response like word reading does. In an inhibitory discussion, this conflict could be seen as requiring interference control to minimize the distractor influence. The second conflict arises when the wrong colored bar is the same color as the Stroop color—both irrelevant to the task. The comparison between colors happens automatically and triggers an erroneous prepotent response that should be inhibited so participants can choose the correct response (Dittrich and Stahl 2016). This is called the Relationship Effect because the wrong colored bar is related to the wrong Stroop attribute. Evidence that the Relationship Effect involves the trigger of a fast and wrong response is that it is characterized mainly by a high number of commission errors and not necessarily slower response times (Afonso Jr et al. 2020; Machado‐Pinheiro et al. 2024). Therefore, inhibition of prepotent responses is necessary to overcome the Relationship Effect.

To investigate the cancellation of initiated responses and how it interacts with other cognitive demands, some studies add stop‐signals to different paradigms (e.g., Khng and Lee 2014). This has been done to inhibitory tasks such as the flanker and the traditional Stroop task (Verbruggen et al. 2004). In these studies, a visual or auditory stop‐signal infrequently appears during the task indicating that the response to the primary task should be suppressed. The results of these studies revealed that the stop‐signal demands can be observed in neutral or congruent conditions and also interact with other inhibitory demands of the task in incongruent conditions. Therefore, this design seems fit to study suppression of ongoing responses in inhibitory paradigms. The stop‐signal has also been combined to the Stroop‐matching task and showed the same pattern of interaction between stopping demands and the conflicts inherent to the task (Afonso Jr et al. 2021).

The Stroop‐matching/stop‐signal task not only allows one to distinguish between different forms of inhibitory processes by means of specific comparisons (i.e., Congruency Effect = interference control; Relationship Effect = inhibition of prepotent responses; Stop‐signals = suppression of ongoing responses) but it also does so using a single task. This is very useful because it reduces task impurity problems common in the assessment of cognitive control functions. Task impurity refers to the fact that every inhibitory task demands the inhibition of some specific kind of response or stimuli. Thus, the measure obtained is not of pure inhibitory processes because it is contaminated by the idiosyncrasies of the task, such as language, visuospatial and responses required (Friedman and Miyake 2004; Miyake et al. 2000). As a result, data from inhibitory tasks also reflect variation in other lower‐level processes necessary for task resolution. Comparing conditions of the same task allows one to subtract out the commonalities of lower‐level processes, reducing impurity (Friedman and Banich 2019). In this sense, the Stroop‐matching/stop‐signal task can be used to get a purer measure of the three inhibitory processes than studies using different tasks for each inhibition.

### 1.4 Objective

The current study investigated three different inhibitory processes using a Stroop‐matching/stop‐signal task. The Congruency Effect reflects interference control demands, Relationship Effect reflects inhibition of prepotent responses and stop‐signal trials demand the suppression of ongoing responses. The advantage of using a single protocol is to diminishes impurity effects caused by irrelevant demands inherent to different tasks. Using this protocol, the main objective of this study is to investigate brain activities associated with each inhibitory function using functional near‐infrared spectroscopy (fNIRS). FNIRS is a functional neuroimaging technique that uses near‐infrared light to measure the concentration of hemoglobin in target brain areas. So, it provides an indirect measure of cortical activity that can elucidate the involvement of brain regions associated with different forms of inhibition. A previous short‐communication from our group was published using the same protocol to investigate the association between inhibitory activities and attention deficit hyperactivity disorder (ADHD) symptoms (Afonso Jr et al. 2023). That first study had a smaller sample size and thus compensated by performing fNIRS analyses over brain regions of interests (ROI) to maximize statistical power (Poldrack 2006). However, ROI analyses also have some drawbacks. Importantly, the regions are arbitrarily created from the aggregation of specific channels and may result in loss of information from averaging channels (Stanley et al. 2013). Therefore, the current study intended to focus on exploring the brain regions associated with different kinds of inhibition by analyzing hemodynamic changes at specific channels rather than at predetermined ROIs. Our hypothesis is that frontal and parietal activities will be differently associated with each kind of inhibition, especially in the DLPFC, IFC, IPS and TPJ, thus supporting the diversity of inhibitory processes and yielding their neural bases during the Stroop‐matching/stop‐signal task.

## 2 Materials and Methods

### 2.1 Participants

Participants were initially 63 university students (14 men), aged between 18 and 28 years (*M* = 21.5; SD = 3.41), but 11 participants were excluded due to poor quality fNIRS data. The final sample size was 52 (10 men, mean age = 21.4; SD = 3.44). Power analysis considering the ANOVA test, alpha = 0.05, beta = 0.80, and an *f* effect size = 0.25 shows that this is a reasonable sample size. They had normal or corrected‐to‐normal visual acuity and normal color vision according to Ishihara's test for color blindness (Ishihara 1972). They were all right‐handed according to the Edinburgh Handedness Inventory—Short Form (Veale 2014). This study was part of a bigger project that aimed to investigate neurocognitive functioning in relation to the dimensional perspective of attention deficit hyperactivity disorder (ADHD). The diagnosis of ADHD was not an inclusion criterion. Participants were excluded if they presented other psychiatric or neurodevelopmental disorders. No participant was excluded for this reason. All procedures were approved by the Research Ethics Committee of Mackenzie Presbyterian University (CEP/UPM; no. 37336720.8.0000.0084) and written informed consent was obtained before the study. The work was carried out in accordance with The Code of Ethics of the World Medical Association (Declaration of Helsinki).

### 2.2 Equipment and Procedure

The experiment was conducted in a sound‐attenuated room with dim ambient light. Participants sat in front of an LCD monitor (with a resolution of 1280 × 768 pixels) at a distance of approximately 57 cm from the screen. At this distance, 1 cm on the screen corresponds to 1° of visual angle. A microcomputer running E‐Prime v.2.0 (Psychological Software Tools, Sharpsburg, PA) timed the stimuli presentation and recorded key presses. A keyboard was placed in front of the participants, and the keys “Z” and “M” were used as response keys and were operated by the left and right index fingers, respectively.

The adequate fNIRS cap was chosen according to each participant head size. The fNIRS cap was then placed in their heads and the optodes were inserted in their respectively cap holes by the researchers. Hair that was obstructing the way between optode and scalp was removed to improve the quality of signal. Data collection also included electromyography (EMG) registration of activity on both hands and an elbow, but the EMG data was not included of the current study. Only then the experiment began. The fNIRS setting and the experiment lasted approximately 40 min each.

#### 2.2.1 Stroop‐Matching/Stop‐Signal Task

**FIGURE 1:**
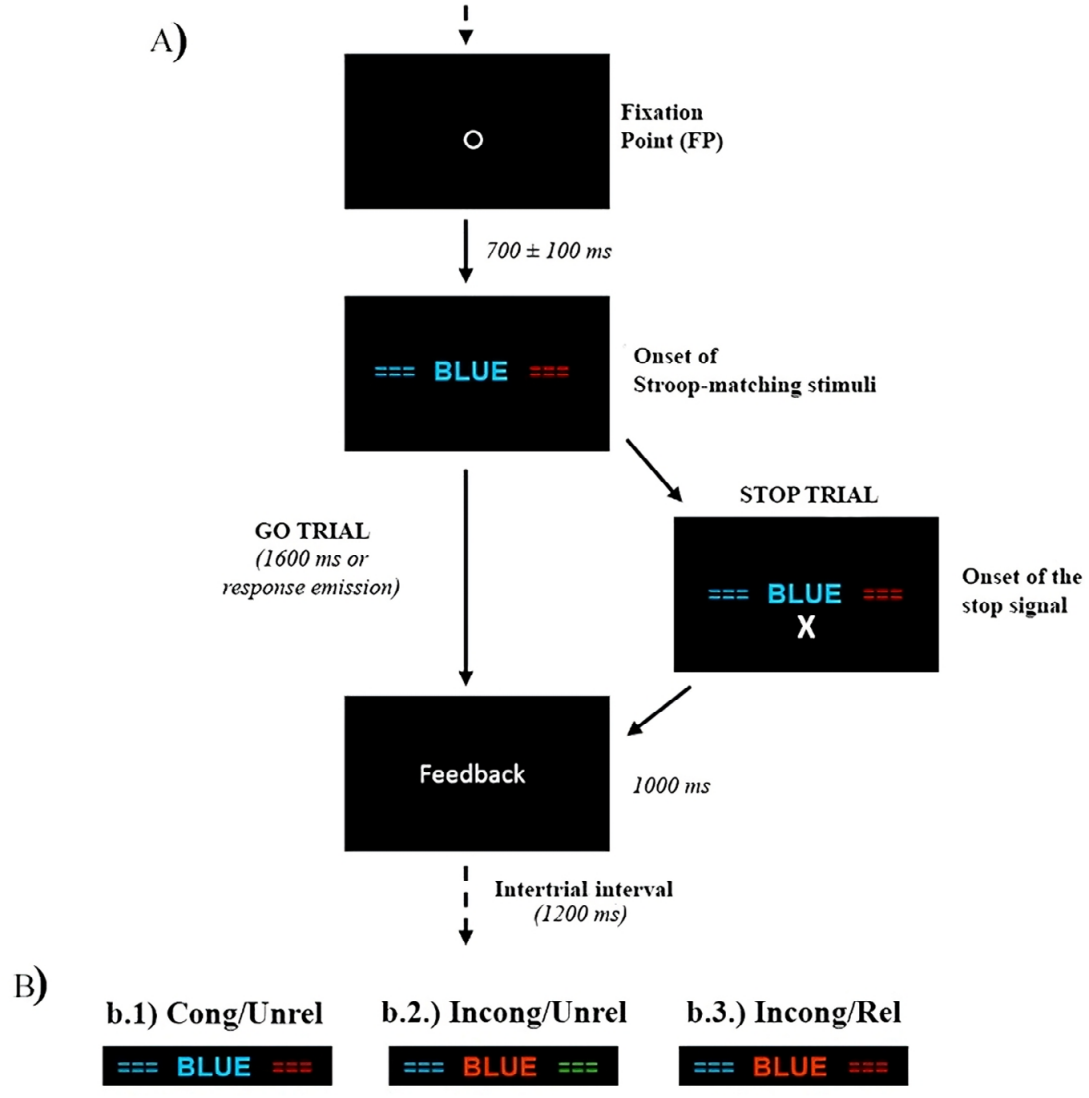
(A) Schematic representation of stimulus display and temporal sequence of the Stroop‐matching/stop‐signal task for go and stop trials. Participants had to compare the central Stroop‐word with the two peripheral bars and press the corresponding spatial key. The first set of blocks only included go trials, whilst the second set also included 1/3 of stop trials; (B) Summary of the three Stroop‐matching conditions adopted. In the scheme, the correct responses are always on the left: A blue bar in response to the word “BLUE”: (b.1) Congruent/unrelated; (b.2) Incongruent/unrelated; (b.3) Incongruent/related. Blocks could be either single‐condition, presenting only one of these conditions in sequence, or mixed‐conditions. The order of the blocks was pseudorandomized, but only the single‐condition blocks were analyzed in this study.

The version of the Stroop‐matching/stop‐signal task adopted here was developed by our group and was used in previous studies (Machado‐Pinheiro et al. 2024; Afonso Jr et al. 2020; Portugal et al. 2018). The experiment was adapted into a block‐design structure to accommodate the requirements of the fNIRS measure. The task is a combination of the Stroop‐matching and stop‐signal tasks—see Figure 1A. The Stroop‐matching task was employed as the primary task, during which stop‐signals were occasionally presented. Each trial began with a fixation point (FP; a white empty circle with 0.3° of visual angle against a black back‐ground) presented for 700 ± 100 ms. Next, three stimuli were presented simultaneously: a Stroop stimulus and two colored bars. The Stroop stimulus appeared centrally, in the place of the FP, and the two colored bars (3.5° × 1.0° of visual angle) appeared laterally to it, on its left and right side. The distance from the center of the Stroop stimulus to the center of the bars was 7.2°. Each letter in the Stroop stimulus had 0.5° of visual angle. In non‐stop trials, the colored bars and the Stroop stimulus remained on the screen for 1600 ms or until a manual response was executed. In stop trials (right side of Figure 1), the stop signal (a white “X”) appeared 1.5° below the Stroop stimulus. Participants were instructed to match the meaning of the Stroop word to the colored bars and to press the spatially corresponding key as fast and accurately as possible—reaction time (RT). Thus, the colored bars should be viewed as “response options”

The Stroop stimuli were composed by the words red, green and blue, printed in red, green, or blue colors. The lateral bars could also be colored in red, green or blue. Participants had to choose which colored bar matched the meaning of the Stroop word, while ignoring the color of the Stroop stimulus and the wrong colored bar. Three conditions arise from the possible combinations of Stroop and bars attributes. First, the Stroop attributes could be congruent (e.g., the word “blue” colored in blue—[b.1]) or incongruent (e.g., the word “blue” colored in red or green—[b.2] and [b.3]). Moreover, the bar that represented the wrong response could match or mismatch the irrelevant Stroop attribute (i.e., the color). The related condition is the one in which the wrong bar matched to the Stroop color (b.3), whereas the unrelated conditions are those in which the wrong bar mismatched to the Stroop color (b.1, b.2). Therefore, our task encompassed three conditions—see Figure 1B: (b.1) Congruent/Unrelated (Cong/Unrel), (b.2) Incongruent/Unrelated (Incong/Unrel), and (b.3) Incongruent/Related (Incong/Rel). The congruency effect is the difference between Incong/Unrel and Cong/Unrel conditions, assuming a subtractive logic that the difference between them is due to the incongruency of the Stroop stimulus. In the current framework, the congruency effect reflects the interference control mechanisms responsible for inhibiting the distractive information of the color of the Stroop stimulus. The relationship effect also assumes a subtractive logic between the Incong/Rel and Incong/Unrel, whose difference lies in the relatedness of the incorrect response option. It involves inhibition of a fast prepotent response generated by the automatic matching between the colors of the Stroop stimulus and the wrong colored bar (i.e., inhibition of prepotent responses).

In stop trials, the stop‐signal appeared after a fixed range of intervals that were set following the procedure employed in the fNIRS study of Ishii‐Takahashi et al. (2014). Thus, the stop‐signal could be presented after three possible intervals: (1) the mean RT in that condition; (2) the mean RT minus 100 ms; and (3) the mean RT minus 250 ms. These intervals were calculated individually for each Stroop‐matching condition. Participants were instructed not to respond when the stop‐signal appeared. Finally, it was emphasized that they should not wait for the appearance of the stop‐signal because it would only occur in a minority of trials. The stop‐signal demand is directly associated with the suppression of ongoing responses.

Feedback was given after each trial. In non‐stop trials, RTs shorter than 150 ms or longer than 1600 ms were considered errors, and the messages “anticipation” or “slow response,” respectively, were presented on the screen. If the participant pressed a key on a stop‐signal trial, the message “stop task” appeared to indicate that it was a stop trial and keys should not be pressed. When the participant pressed the wrong key, the message “incorrect” appeared. Finally, the message “correct” was shown if the response was accurate in a non‐stop trial, if the response was correctly inhibited in a stop trial, or if the participant pressed the correct key before the stop‐signal presentation in a stop trial. Feedback messages remained on screen for 1000 ms, and a new trial began after an additional intertrial interval of 1200 ms.

The task was divided into two sets of blocks. The first set only included non‐stop trials. This set used single‐condition blocks (i.e., the same Stroop‐matching condition in the entire block) and mixed‐conditions blocks (i.e., blocks with trials from all three Stroop‐matching conditions randomized). There were 3 single‐condition blocks, with 12 trials for each Stroop‐matching condition (a total of 9 blocks). There were also 3 mixed‐condition blocks with 12 trials (i.e., 6 Cong/Unrel, 3 Incong/Unrel and 3 Incong/Rel trials per block). The single and mixed‐conditions blocks were distributed in a pseudorandomized way. The mixed‐conditions measures were not analyzed in this study. Their inclusion was strategic to avoid the experiment being easily predictable, since the participants could not foretell whether a block was composed of single or mixed conditions. This first set was preceded by a 26‐trial practice block.

The second set of blocks introduced stop trials. They were composed of 3 single‐condition blocks to each Stroop‐matching condition (a total of 9 blocks). Each block had 18 trials of which 6 (1/3 of total trials) were stop trials. The order of trials was pseudorandomized so that stop‐signal trials would never appear in sequence or before the third trial. Stop‐signal intervals were calculated using the mean RT collected in the first set of Stroop‐matching blocks, as previously described. The adherence to the stop‐signal instructions was assessed by the probability of inhibit (p(inhibit)), that is, the percentage of trials in which the response was correctly interrupted after the stop‐signal. The second set of blocks was also preceded by a 27‐trial practice block including 9 stop‐signal trials.

#### 2.2.2 fNIRS

**FIGURE 2:**
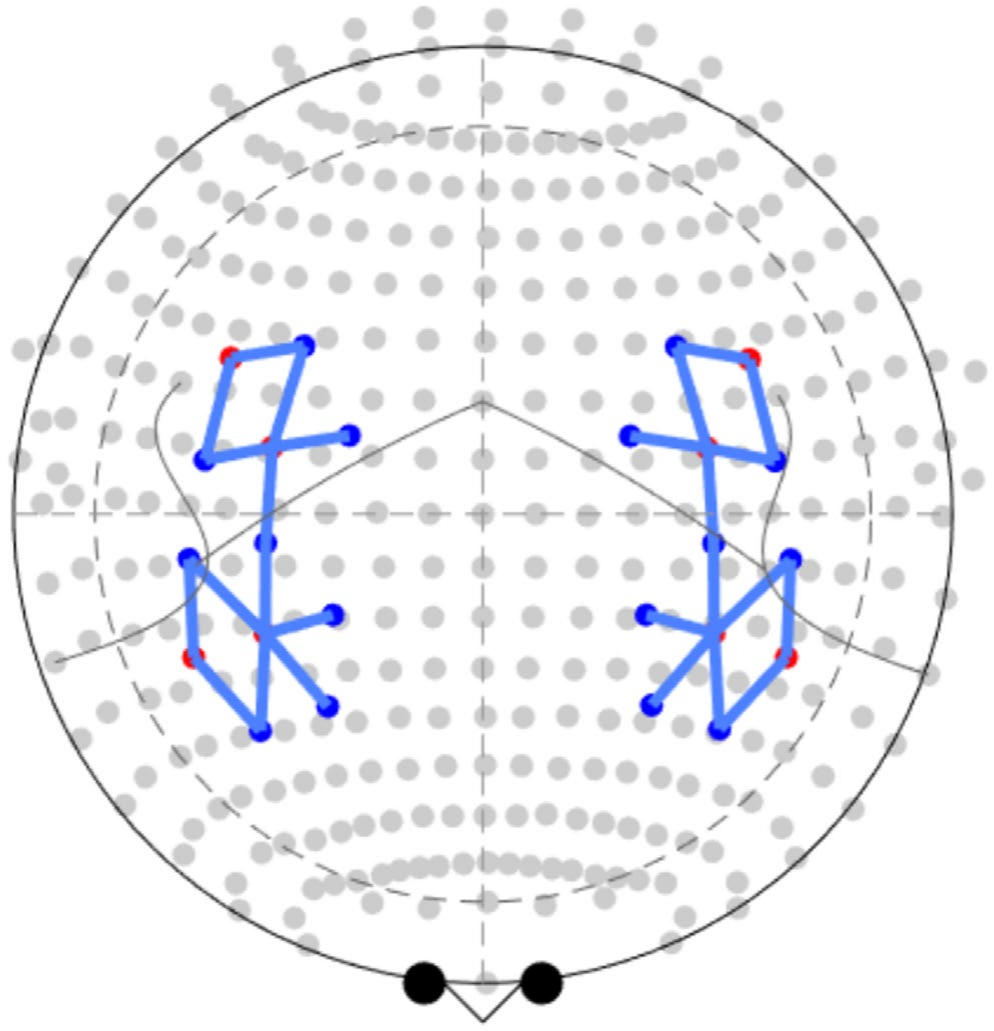
Source‐detector layout and the 26 channels created. Sources are red circles and detectors are blue circles.

Hemodynamic activities during the Stroop‐matching/stop‐signal task were acquired by a multi‐channel, continuous wave, fNIRS system (BrainSight, Rogue Research Inc., Canada). It comprised 8 source optodes (laser diodes) that emitted light at two near‐infrared wavelengths (705 and 830 nm) and 16 detector optodes (avalanche photodiodes, APDs). The optodes were organized in such a way that the final probe consisted of 26 measurement channels covering frontal and temporoparietal regions (Figure 2). Each pair of source‐detector that made up a channel was separated by approximately 3 cm. The position of each optode was chosen according to the MNI coordinates associated with each hole in the cap (BrainSight, Rogue Research Inc., Canada). The MNI coordinates from the optodes used are listed in Table 1. Participants had the circumference of their heads measured in the beginning of the experiment as well as the distance between inion‐nasion and right–left ear canals in order to choose the best fitting cap and also to standardize the position of the cap across the participants. Data were collected using a sampling rate of 10 Hz.

**TABLE 1:** Distribution of sources and detectors and their respective MNI coordinates.

	*x*	*y*	*z*
*Frontal regions*			
Left hemisphere			
Source 1 (S1)	−66.651	16.4933	30.5043
Source 2 (S2)	−73.8372	19.8173	−4.1777
Detector 1 (D1)	−50.7785	40.2739	44.7777
Detector 2 (D2)	−81.5364	−5.0503	12.6896
Detector 3 (D3)	−62.3835	41.2781	12.2637
Detector 4 (D4)	−52.1191	12.3691	60.9188
Right hemisphere			
Source 5 (S5)	67.2775	16.5879	29.4005
Source 6 (S6)	73.9741	19.7221	−5.3125
Detector 9 (D9)	81.8881	−5.177	11.5727
Detector 10 (D10)	62.6143	41.4882	10.9904
Detector 11 (D11)	53.0709	12.3551	59.9441
Detector 12 (D12)	51.496	40.4943	43.5318
*Temporoparietal regions*			
Left hemisphere			
Source 3 (S3)	−68.7071	−41.3767	57.314
Source 4 (S4)	−74.9402	−63.5092	33.3628
Detector 5 (D5)	−58.222	−72.8335	61.2648
Detector 6 (D6)	−46.948	−47.9687	79.9337
Detector 7 (D7)	−81.2707	−33.5182	26.1154
Detector 8 (D8)	−69.7443	−10.9153	45.5941
Right hemisphere			
Source 7 (S7)	69.4084	−41.5163	56.4805
Source 8 (S8)	75.24	−63.7483	32.5475
Detector 13 (D13)	58.752	−72.9431	60.6761
Detector 14 (D14)	47.8242	−48.0445	79.4678
Detector 15 (D15)	81.6435	−33.7056	25.0981
Detector 16 (D16)	70.5157	−10.958	44.6378

### 2.3 Analysis

Reaction time (RT) and accuracy analyses were performed only for the single‐condition blocks of the Stroop‐matching task. Non‐stop and stop‐signal blocks were analyzed separately. Mean RTs of correct trials, the percentage of incorrect responses, and p(inhibit) were submitted to individual repeated measures analysis of variance (ANOVA) using condition (Cong/Unrel, Incong/Unrel, and Incong/Rel) as a within‐subject factor. The partial eta‐squared ($\eta_{p}^{2}$) was calculated as a measure of effect size. Post hoc tests were calculated using the Newman–Keuls test.

Analyses of fNIRS data were performed using functions from the NIRS Brain AnalyzIR (Santosa et al. 2018) and the Homer 3 (Huppert et al. 2009) toolboxes. Following a block‐design, hemodynamic responses were analyzed per block. Hemodynamic activity in Stroop‐matching blocks without stop‐signals were analyzed for the first 30 s of the task, whereas blocks with stop‐signals were analyzed for the initial 60 s. Only oxygenated hemoglobin (HbO) signals were analyzed due to its higher reliability (Schecklmann et al. 2008), better signal‐to‐noise ratio and stronger association with the BOLD signal in fMRI analyses (Cui et al. 2011). Signal quality was checked using the hmrPruneChannels function of the Homer toolbox in which bad channels were identified and excluded according to the quotient between mean and standard deviation of the signal (SNR = 2). Participants that presented 10 or more bad channels were dropped from the experiment. Eleven participants were excluded this way. Initially, data were resampled to 5 Hz. The raw light signal was converted to optical density and then to HbO concentration estimates through the modified Beer–Lambert law. In the first‐level of analysis, individual task activations were calculated using the general linear model (GLM). The canonical hemodynamic response function (HRF) was used as model to predict a hemodynamic response during the period of each block, assuming the linear and time invariant summation of overlapping hemodynamic responses (Plichta et al. 2007). Coefficients (i.e., *β*) for each channel and condition of the task were estimated using the autoregressive iteratively‐reweighted least squares (AR‐IRLS) approach to solve the general linear equation. AR‐IRLS already accounts for serial correlations in the data, including systemic physiological noise and motion artifacts (Barker et al. 2013). Thefore, *β* are estimates of the average change in HbO for each channel.

In the second‐level analysis, linear mixed‐effects models were used to calculate activation at group‐level using the *β*‐values estimated at the first level for each channel using condition as independent variables and the subjects as a random effect. Then, contrasts were performed using the group‐level data and student's *t*‐tests were used to compare the mixed model coefficients between conditions. The three contrasts were designed to isolate the forms of inhibition of our interest: 1. Congruency Effect (Incong/Unrel—Cong/Unrel); 2. Relationship Effect (Incong/Rel—Incong/Unrel); and 3.1. Stop‐signal (Cong/Unrel/Stop‐signal blocks—Cong/Unrel/Non‐stop‐signal blocks); 3.2. Stop‐signal/Congruency Effect (Incong/Unrel/Stop‐signal blocks—Incong/Unrel/Non‐stop‐signal blocks); 3.3. Stop‐signal/Relationship Effect (Incong/Rel/Stop‐signal blocks—Incong/Rel/Non‐stop‐signal blocks). Thus, the Congruency Effect contrast reflects activity associated with interference control; the relationship effect contrast reflects inhibition of prepotent responses demands; and the Stop‐signal contrast reflects activity associated with (3.1.) suppression of ongoing responses and its interaction with (3.2.) interference control and (3.3.) inhibition of prepotent responses.

All analyses adopted an α‐level of 0.05 for statistical significance. For fNIRS analyses, false discovery rate (FDR) corrections to control for multiple comparison were applied, setting the threshold of the corrected *p* value (i.e., *q* value) to 0.05.

## 3 Results

### 3.1 Stroop‐Matching Task (Non‐Stop Blocks)

The RT ANOVA showed a main effect of condition (*F*(2,102) = 219.42, *p* < 0.001, $\eta_{p}^{2}$ = 0.82). The post hoc analysis revealed that the mean RT in the congruent condition (Cong/Unrel = 554 ms) was faster than the two incongruent conditions (Incong/Unrel = 651 ms; Incong/Rel = 682 ms, *p* < 0.001 for both). Incong/Rel was significantly slower than Incong/Unrel (*p* < 0.001). The error rate ANOVA replicated the RT results. It showed a main effect of condition (*F*(2,102) = 19.72, *p* < 0.001, $\eta_{p}^{2}$ = 0.28). The post hoc analysis revealed that the Cong/Unrel (0.4%) had lower error rate than Incong/Unrel (2.03%, *p* = 0.006) and Incong/Rel (4.00%, *p* < 0.001). Incong/Rel also had a higher error rate than Incong/Unrel (*p* < 0.001). These results indicated that the congruency effect and relationship effect were present in both RT and accuracy in the Stroop‐matching task during blocks without stop‐signals. Therefore, the commonly reported effects found in the Stroop‐matching task are still present in the current experiment despite of the block‐design adopted. RT and accuracy descriptive data for the Stroop‐matching task (non‐stop blocks) are summarized in Table 2.

**TABLE 2:** Mean and standard deviation of reaction times, error rate, and probability of inhibit in the Stroop‐matching task (stop and non‐stop blocks).

	Reaction time		Error rate		Probability of inhibit	
	Mean	SD	Mean	SD	Mean	SD
Stroop‐matching task (non‐stop blocks)						
Cong/unrel	554	109	0.40	2.97	—	—
Incong/unrel	651	113	2.03	3.57	—	—
Incong/rel	682	128	4.00	6.51	—	—
Stroop‐matching task (stop blocks)						
Cong/unrel	536	92	1.35	3.11	0.46	0.24
Incong/unrel	642	103	4.02	4.62	0.36	0.19
Incong/rel	661	103	5.20	5.61	0.34	0.21

### 3.2 Stroop‐Matching/Stop‐Signal Task (Only Stop Blocks)

When considering only blocks that included stop‐signal trials, the RT ANOVA showed a main effect of condition (*F*(2,102) = 200.81, *p* < 0.001, $\eta_{p}^{2}$ = 0.79). Again, Cong/Unrel (536 ms) was faster than Incong/Unrel (642 ms, *p* < 0.001) and Incong/Rel (661 ms, *p* < 0.001), and the two incongruent conditions differed (*p* = 0.005). The error rate ANOVA showed a main effect of condition (*F*(2,102) = 17.72, *p* < 0.001, $\eta_{p}^{2}$ = 0.24). The post hoc revealed that Cong/Unrel (1.35%) had a lower error rate than Incong/Unrel (4.02%, *p* < 0.001) and Incong/Rel (5.20%, *p* < 0.001). Contrary to the ANOVA with non‐stop blocks, the two incongruent conditions did not show a significant difference in their error rate (*p* = 0.09). Lastly, the p(inhibit) ANOVA showed that Cong/Unrel (0.46) had a higher probability of inhibiting responses than Incong/Unrel (0.36, *p* < 0.001) and Incong/Rel (0.34, *p* < 0.001) whereas the two latter did not differ significantly. Therefore, in blocks with stop‐signal, the congruency effect was apparent in RT, error rate, and p(inhibit), while the relationship effect was only shown in RT. Nevertheless, the Cong/Unrel condition showed faster RT, lower error rate, and higher probability of inhibiting than the two incongruent conditions. RT, accuracy, and p(inhibit) descriptive data for the Stroop‐matching task (stop blocks) are summarized in Table 2.

### 3.3 fNIRS Results

**FIGURE 3:**
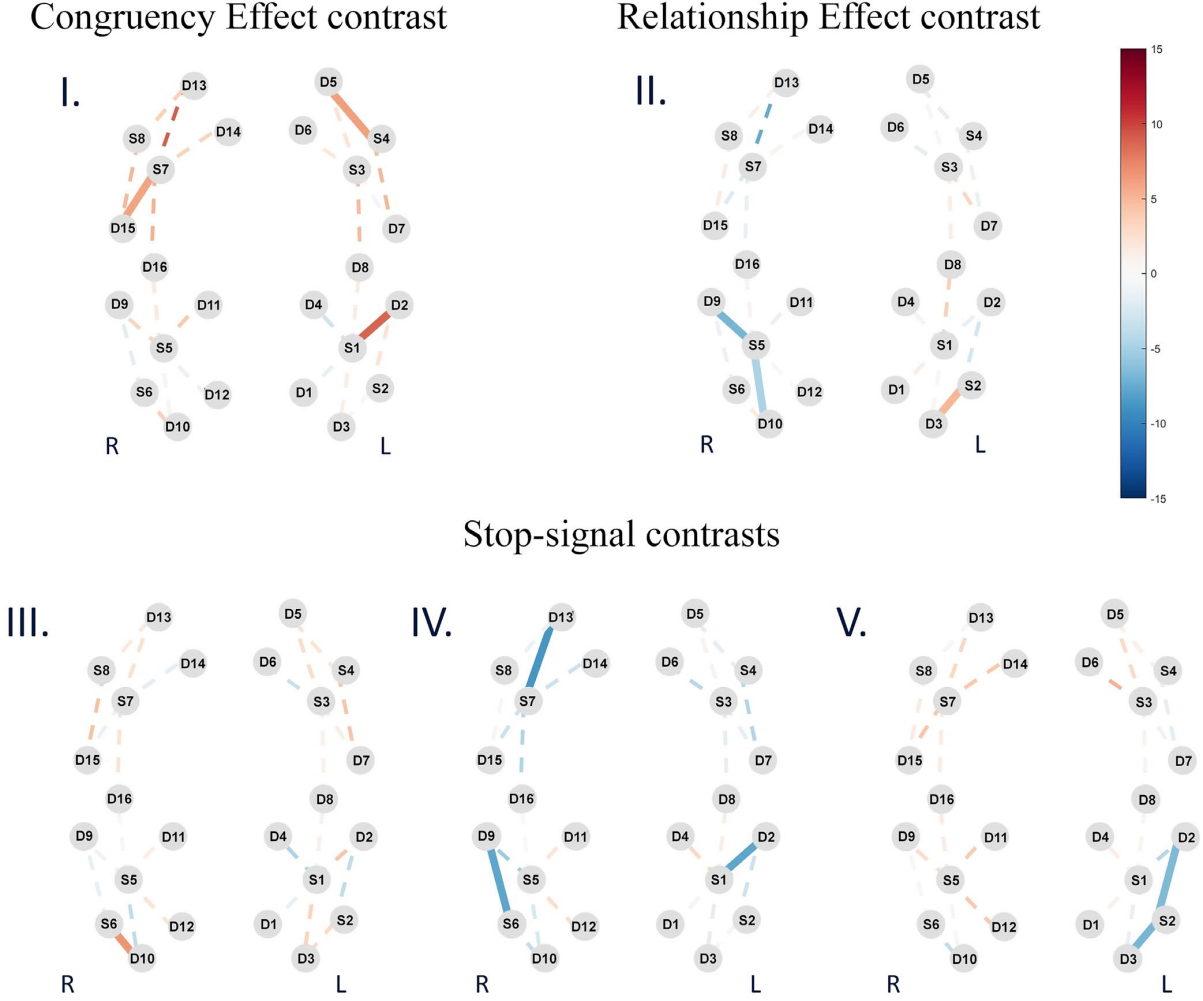
Group‐level results of HbO changes for the three types contrasts adopted: (I) Congruency Effect contrast = Incong/Unrel—Cong/Unrel; (II) Relationship Conflict contrast = Incong/Rel—Incong/Unrel; Stop‐signal contrast includes; (III) Cong/Unrel (stop blocks)—Cong/Unrel (non‐stop blocks) = Stop‐signal blocks—Non‐stop‐signal blocks; (IV) Incong/Unrel (stop blocks)—Incong/Unrel (non‐stop blocks); and (V) Incong/Rel (stop blocks)—Incong/Rel (non‐stop blocks). Activation maps (beta values) with significantly active channels (*q* < 0.05) are shown in solid lines.

The results of the contrasts of brain activities between task conditions are presented in the form of activation maps in Figure 3. In the Congruency Effect contrast (Figure 3I) there was an increased activation in channels over the left IFC (S1‐D2, *β* = 8.87, *t* = 4.96, *q* < 0.001), left IPS (S4‐D5, *β* = 6.36, *t* = 2.94, *q* = 0.03) and right IPS (S7‐D15, *β* = 6.01, *t* = 2.96, *q* = 0.03) in the Incong/Unrel condition, compared to Cong/Unrel. In the Relationship Effect contrast (Figure 3II), there was an increased activation in a channel over the left IFC (S2‐D3, *β* = 5.05, *t* = 3.71, *q* = 0.005) and decreased activation in channels over the right IFC (S5‐D9, *β* = −7.02, *t* = −3.63, *q* = 0.005; and S5‐D10, *β* = −4.78, *t* = −2.93, *q* = 0.03) in the Incong/Rel condition, compared to Incong/Unrel. In the Stop‐signal contrast (Figure 3III), there was an increased activation in a channel over the right IFC (S6‐D10, *β* = 6.75, *t* = 3.51, *q* = 0.01) in Cong/Unrel stop‐signal blocks, compared to Cong/Unrel nonstop‐signal blocks. In the Stop‐signal/Congruency Effect contrast (Figure 3IV), there were decreased activations in the left IFC (S1‐D2, *β* = −7.99, *t* = −3.95, *q* < 0.001), in the right IFC (S5‐D9, *β* = −5.43, *t* = −2.57, *q* = 0.07; S6‐D9, *β* = −7.85, *t* = −3.33, *q* = 0.001) and in the left IPS (S7‐D13, *β* = −8.82, *t* = −3.00, *q* = 0.003) in Incong/Unrel stop‐signal blocks, compared to Incong/Unrel nonstop‐signal blocks. Lastly, the Stop‐signal/Relationship Effect contrast (Figure 3V) showed decreased activation in channels over the left IFC (S2‐D2, *β* = −6.62, *t* = −2.98, *q* = 0.04; S2‐D3, *β* = −6.98, *t* = −4.42, *q* < 0.001) in Incong/Rel stop‐signal blocks, compared to Incong/Rel nonstop‐signal blocks.

## 4 Discussion

This study used fNIRS to investigate how frontal and parietal regions activate according to different inhibitory demands in a combined Stroop‐matching/stop‐signal task. The performance results (i.e., reaction time, accuracy of responses and probability of inhibition) replicated the effects commonly found in the Stroop‐matching/stop‐signal task, i.e., the Incongruent/Related condition showed slower reaction times and worse accuracy than the Incongruent/Unrelated condition, which showed slower reaction times and worse accuracy than the Congruent/Unrelated condition – the Congruency and Relationship Effects. This difference is attributed to the inhibitory demand of each condition (Dittrich and Stahl 2016; Machado‐Pinheiro et al. 2024). Also, the probability of inhibition in stop trials was different in each Stroop‐matching condition, indicating an interaction between Stroop‐matching and stop‐signal mechanisms of inhibition (Portugal et al. 2018; Kalanthroff and Henik 2013; Verbruggen et al. 2004). These performance results confirm that the Stroop‐matching/stop‐signal task was eliciting the usual inhibitory processes suggested by the literature and could therefore be used to analyze brain functions in response to the different inhibitory demands.

### 4.1 Congruency Effect Contrast

The Congruency Effect contrast showed increased activity in a channel over a mid‐posterior region of the left IFC. Functional neuroimaging findings revealed that Stroop tasks, in comparison to other inhibitory paradigms such as flanker, go/no‐go, stimulus–response compatibility, Simon, and stop‐signal tasks, specifically show activation of inferior frontal regions (as well as dorsolateral regions) and that these activations are highly left lateralized (Nee et al. 2007), which was hypothesized to be due to the verbal conflict inherent to the task (Leung et al. 2000). In fact, a recent activation likelihood estimation (ALE) meta‐analysis focusing on studies with young adults showed that activity in the left IFG is frequently found in neuroimaging studies that contrast incongruent with congruent Stroop conditions (Huang et al. 2020), such as the present one. Moreover, several studies described the role of the left IFC as preventing interference from the competition of relevant and irrelevant stimulus or representations, that is, interference control. For example, IFC activity in incongruent trials is consistent with the Cascade‐of‐Control model, which posits that interference in a Stroop task can occur at multiple stages, from stimulus input to response selection (Banich 2019). In this model, the posterior portion of bilateral IFC is responsible for biasing activity in favor of the most task‐relevant information. Other regions would then select information in working memory (DLPFC), select the appropriate response (caudal mid‐cingulate) and evaluate the response (rostral dorsal anterior cingulate cortex) (Banich 2019). In a similar vein, studies about cognitive control in language processing suggested that the left IFC implements control in the face of representational conflict by biasing the activation of relevant representations in order to prevent errors (Novick et al. 2005). This kind of role is supported by a study focusing on the cognitive control of memory that argued that the anterior part of the left inferior frontal gyrus would control access to stored representations in memory while a left mid‐inferior frontal gyrus would act in resolving competition among active representations (Badre and Wagner 2007). These studies highlight the role of mid and posterior regions of the left IFC in solving competition among relevant and irrelevant representations. This evidence indicates that the left IFC is recruited in the Stroop‐matching task in order to implement the interference control required in incongruent trials, where the difference between color and word of the Stroop stimulus hinders performance (i.e., the congruency effect seen in reaction times and accuracy).

The Congruency Effect contrast also yielded significant activation of bilateral channels over the IPS. More recently, a range of studies has suggested that the posterior parietal cortex (PPC), including the IPS, has an important role in cognitive control (Kolodny et al. 2017; Osada et al. 2019). This could be at least partially explained by the reciprocal projections the PPC maintains with PFC regions associated with inhibition (Fox and Raichle 2007). The PPC, considered an important node in the fronto‐parietal network (Friedman and Robbins 2022; Menon and D'Esposito 2022) is associated with attention and working‐memory functions (Katsuki and Constantinidis 2012), and specifically with handling distractors (Friedman‐Hill et al. 2003). The IPS, part of the PPC, integrates task‐relevant information from different modalities (Gottlieb 2007) and shows increased activity when distractors are presented alongside target stimuli (Chun and Marois 2022; Marois et al. 2000). Therefore, the IPS has already been attributed the function of interference control (Zysset et al. 2001). Our finding that bilateral IPS was active in the Congruency Effect contrast, but not in the Relationship Effect contrast, supports the idea that these parietal regions are important to the inhibition of distractive information.

### 4.2 Relationship Effect Contrast

The Relationship Effect contrast, as the Congruency Effect contrast, showed an increased activation in the left IFC, albeit in a more anterior channel. The difference between the Incong/Rel and Incong/Unrel conditions demonstrated in the Relationship Effect contrast is regarded as being due to an additional demand of inhibiting a wrong prepotent response that is elicited automatically by an unnecessary comparison between Stroop color and wrong colored bar (Afonso Jr et al. 2021; Dittrich and Stahl 2016). The IFC is frequently linked to the inhibition of motor functions (Aron et al. 2014), but lateralization is a crucial aspect of the kind of behavioral inhibition that it performs. It is well supported by previous studies that the right IFC is active in tasks that require suppression of ongoing responses, such as the stop‐signal tasks (Boen et al. 2022), whereas the left IFC has been shown to be involved in the inhibition of prepotent responses in a lesser extend (Swick and Chatham 2014). Considering that inhibition of prepotent responses and suppression of ongoing responses are two different facets of motor inhibition (Raud et al. 2020), it is expected that each one shows unique neural bases. The current study contributes to the incipient body of evidence that argues in favor of a left IFC role in the inhibition of prepotent responses, further advocating for the dissociation between different forms of motor inhibition.

Additionally, Incong/Rel revealed a decrease in activity in comparison to Incong/Unrel in the right IFC, indicating that the latter condition engages the right IFC more than the former. Notably, there was no significant difference in activation of this channel between Cong/Unrel and Incong/Unrel conditions following the Congruency Effect contrast. Together, these results suggest that the right IFC is more active in both unrelated conditions than in the related one. The right IFC is usually mentioned as an important region to inhibit actions due to its strong connections to motor regions like the presupplementary motor area and motor circuits via the subthalamic nucleus (Aron et al. 2014). However, it seems unlikely that any brain deactivation in the Relationship Effect contrast reflects diminishment of inhibitory demands, since Incong/Rel conditions include the same inhibitory demands as Incong/Unrel (and Cong/Unrel, for that matter) and some more. An alternative explanation lies in the finding that the right IFC is also associated with other non‐inhibitory demands even in inhibitory paradigms (Kolodny et al. 2017). A greater IFC activity in unrelated conditions can be interpreted as evidence of the function of a multiple‐demand network that shows domain‐general activations across cognitive tasks (Camilleri et al. 2018). This multiple‐demand network, of which the IFC is part is responsible for a set of behavioral and cognitive functions, including not only inhibition but also attention, alertness, and working memory, for example (Camilleri et al. 2018; Kaufmann et al. 2023). Thus, Incong/Unrel and Cong/Unrel conditions could require non‐inhibitory abilities that Incong/Rel does not. Similarly, a multiple‐demand perspective could also explain the earlier finding showing that the left IFC is involved both in interference control (Congruency Effect contrast) and inhibition of prepotent response (Relationship Effect contrast). In this scenario, the left IFC would not have a specific function; instead, it would participate in multiple functions related to cognitive control and would then be active across different tasks. However, the current study was not designed to answer these questions, and more studies are necessary to understand the role of the IFC in the Stroop‐matching task. Studies contrasting conditions that do not vary in inhibitory demand, but in other demands, such as attentional, working memory, or response/stimulus settings, could elucidate this issue.

### 4.3 Stop‐Signal Contrasts

The objectives of the Stop‐signal contrasts were twofold: (1) Analyzing areas involved in the suppression of ongoing responses in our task (i.e., the Stop‐signal contrast) and (2) analyzing brain function in the interaction between interference control and suppression of ongoing responses (i.e., the Stop‐signal/Congruency Effect contrast) and between inhibition of prepotent responses and suppression of ongoing responses (i.e., the Stop‐signal/Relationship Effect contrast).

#### 4.3.1 Stop‐Signal Contrast

The Stop‐signal contrast showed an increased activation in the right IFC. Importantly, this analysis contrasted activity in Cong/Unrel blocks without stop‐signals with activity in Cong/Unrel blocks with stop‐signal. In the current task, Cong/Unrel is used as a baseline condition in which the inhibitory demand (the interference control) is virtually absent. Therefore, including stop‐signals in this condition is a way to investigate the effect of the stop‐signal demand alone, that is, the suppressing of ongoing responses with no interaction with other inhibitory demands. Our results support the idea that this contrast is revealing activity related to the suppression of ongoing responses, since the right IFC is the frontal region generally associated with this form of inhibition (Suda et al. 2020). The right IFC activity during Cong/Unrel trials with stop‐signal was greater than during Cong/Unrel trials without stop‐signal. Notably, this was exclusive to the right hemisphere, further showing the importance of the lateralization of inhibitory functions. The role of the right IFC in the suppression of ongoing responses has been contested recently (Kolodny et al. 2017; Thunberg et al. 2020) and alternative functions of this region have been proposed, as mentioned in previous paragraphs (i.e., part of a multiple demand network). However, it is more likely that IFC activity is task‐dependent (Simmonds et al. 2008), which would explain such variability in results. In our task, this region seems to act favoring the inhibition of initiated actions. Another possible explanation for different findings concerning the inhibitory function of the right IFC regards individual differences in its organization. Suda and colleagues (2020) evaluated the function of different parcels of the right IFC using precision functional mapping. Their results revealed six modules in the right IFC that had specific correlations with response inhibition and, importantly, whose spatial organization varied considerably across individuals. The authors proposed that individual approaches should be used in the study of the right IFC role in inhibition. Moreover, the parcellation of the IFC in subregions functionally distinct reinforces the channel‐wise analyses used in this study, since ROI analyses could mask this kind of regional specificity.

#### 4.3.2 Stop‐Signal/Congruency Effect and Stop‐Signal/Relationship Effect Contrasts

Finally, the other two stop‐signal contrasts were done in order to investigate the interaction between suppression of ongoing responses and interference control/inhibition of prepotent responses. These two contrasts showed an overall similar pattern, that is, in stop‐signal blocks, channels over areas that were activated in the Congruency Effect contrast (i.e., right parietal and left frontal regions) and Relationship Effect contrast (i.e., left frontal regions) yield decreased activities. This result is similar to the one found in a previous study from our group using the same Stroop‐matching/stop‐signal task (Afonso Jr et al. 2023), but it expands previous conclusions by using a different analyses method. More specifically, in Afonso Jr et al. (2023), a single stop‐signal contrast was performed, averaging all non‐stop conditions of the Stroop‐matching task and comparing it with all stop conditions. Also, it analyzed predetermined regions of interests (ROI) instead of individual channels. The current finding shows that this pattern of deactivation is exclusive to conditions where there is an interaction between inhibitory demands (i.e., Incong/Rel stop and Incong/Unrel stop) and does not appear in our baseline condition (i.e., Cong/Unrel stop). Therefore, we can conclude that this deactivation is not due to the stop‐signal demand in general, but only to the interaction between suppression of ongoing responses and other forms of inhibition. This further supports our hypothesis proposed that the demand for the suppression of an already initiated response leads to a deactivation of cognitive control systems engaged in the Incong/Unrel and Incong/Rel conditions, that is, interference control and inhibition of prepotent responses, respectively. In the only condition where no other inhibitory mechanism is required, there was no such deactivation.

Furthermore, if the hypothesis that the stop‐signal contrasts show a deactivation of systems engaged in the primary task is accepted, then this result also supports the role of the right IFC in the Incong/Unrel condition. As discussed previously, the right IFC did not showed significant activation in the Congruency Effect contrast (i.e., when Incong/Unrel was compared to Cong/Unrel) but it showed decreased activity in the Relationship Effect contrast (i.e., when Incong/Rel was compared to Incong/Unrel), which suggests that the right IFC performed a role in Incong/Unrel trials (i.e., it was more active in Incong/Unrel than in Incong/Rel). The Stop‐signal/Congruency Effect contrast also showed deactivation of the right IFC in Incong/Unrel trials with stop‐signals, reinforcing that this area was indeed involved in this condition without stop‐signals. But, since its activity did not differ in comparison to the Cong/Unrel, it is not very likely that it acts controlling interference. One possibility proposed by a study using a Stroop task is that frontal right activity is related to the inhibition of colors, because the right hemisphere is associated with discrimination of color stimuli (Yasumura et al. 2014). Since the Incong/Unrel is the condition that presents more colors in a single display (i.e., two different colored bars and a third color in the Stroop stimulus), the IFC could be recruited to deal with this specific color demand. However, further studies are necessary to understand which role the right IFC has in Incong/Unrel trials.

The current findings give some support to our previous study that analyzed fNIRS activity through ROI analyses (Afonso Jr. et al. 2023), but they also differ in important ways. The frontal activation found here in the Congruency Effect contrast was not present in the previous study; however, the parietal as well as other frontal and parietal activations were present. The Relationship Effect contrast showed deactivation in left frontal channels and activation of the right frontal channels in both studies, even though the specific channels activated were different. The Relationship Effect yielded deactivation in some parietal channels in the previous study, which supports the current conclusion of parietal activity in Incong/Unrel condition in comparison to Incong/Rel. Also, the current result yielded an overall fewer activations in this contrast too. The Stop‐signal contrasts are not directly comparable because different techniques were applied (i.e., in Afonso Jr et al. (2023) we analyzed the mean average of all stop‐signal conditions whereas in here we separated by Stroop‐matching condition), but the two studies showed a general pattern of deactivation of brain regions in stop‐signal blocks. The differences in results could be explained by a couple of factors. First, the difference in analyzing method (i.e., per channel or per ROI). ROI analyses could be misleading because they depend on the averaging of arbitrary channels (Stanley et al. 2013). Second, the sample in Afonso Jr et al. (2023) did not exclude left‐handed or ambidextrous participants, which were two of the twenty‐five participants (i.e., 8%), which could also influence brain activity that has been shown to be highly lateralized, especially in a task that has such a strong verbal demand as the Stroop‐matching task (Capizzi et al. 2017).

Some limitations of the present study should be considered. First, the task design was adapted to allow data collection using fNIRS. The block‐design adopted here is usually not the design adopted in other behavioral experiments, but it was necessary to fNIRS data collection. The most significant difference is the inclusion and analyses of single condition blocks. This could lead to specific strategies that are not transferred to other studies in which conditions were mixed in the block. The inclusion of blocks with multiple conditions tried to minimize predictive strategies and it seemed to have worked, since performance analyzes yielded the effects commonly seen in the task. However, this blocked‐designed showed similar reaction times and accuracy results than previous study, indicating that there was no significant difference in the task demands. Furthermore, the stop‐signal task, in particular, have many variations in the literature (Verbruggen et al. 2019). For example, some use fixed stop‐signal intervals other used dynamic intervals. Also, different outcomes of the stop‐signal task are used, including p(inhibit) but also the time to stop the action (stop‐signal reaction time, SSRT). Moreover, the primary task in which the stop‐signal is included changes from study to study. These variations may result in different demands and processes involved. Therefore, our stop‐signal results should be carefully interpreted in the context of the current task. Secondly, individual trials were not analyzed. Thus, it is hard to define if brain activity would be different in successfully versus failed inhibited trials, and our data mixed both. Since previous studies indicated that stop‐signal trials activate specific brain regions depending on the success of motor inhibition (Li et al. 2006), future studies should investigate this further. Lastly, there are limitations regarding the localization of the brain regions investigated. An inherent difficult of using fNIRS is that its spatial resolution is about 3 cm, that is the recommended distance of optodes to create a channel (Pinti et al. 2018). Some brain areas may be sufficiently close so that a single channel may cover part of them both. Also, brain regions were localized based on the standard MNI (Montreal Neurological Institute and Hospital) coordinates indicated by the manufacturer of the fNIRS caps used. Individual neuroimages or a neuronavigation system would result in a more precise localization of these brain areas.

## 5 Conclusion

Interference control, indexed in the present study to the Congruency Effect, involved the left IFC and bilateral IPS, supporting the lateralized role of the IFC and parietal regions in handling conflict caused by distractor information. Inhibition of prepotent responses, indexed to the Relationship Effect, also showed activation of the left IFC, which suggests that this region is involved in multiple components of inhibition or may act as part of a multiple‐demand network, acting in several functions associated with cognitive control. Importantly, inhibition of prepotent responses did not involve parietal regions as interference control did. Suppression of ongoing responses corroborated the well‐documented finding of right IFC activation. Moreover, interaction between demands of suppression of ongoing responses and the other two inhibitions led to a pattern of deactivation of frontal and parietal regions supposedly involved in each one. This indicates that the combination of stop‐signal and Stroop‐matching tasks, as designed in the present study, results in the suppression of brain function demanded by the Stroop‐matching task in addition to the suppression of motor responses. Notably, we did not find involvement of channels over the DLPFC in any contrast performed. This study corroborates the idea of multiple inhibitory components characterized by unique performance and neurophysiological activities in a context with reduced impurity problems.

## Author Contributions

**Armando dos Santos Afonso Junior:** conceptualization, data curation, formal analysis, funding acquisition, investigation, methodology, project administration, validation, visualization, writing – original draft, writing – review and editing. **Walter Machado‐Pinheiro:** conceptualization, funding acquisition, investigation, methodology, project administration, supervision, validation, visualization, writing – review and editing. **Luiz Renato Rodrigues Carreiro:** conceptualization, funding acquisition, investigation, methodology, project administration, resources, supervision, validation, visualization, writing – review and editing.

## Conflicts of Interest

The authors declare no conflicts of interest.

## Data Availability

The data that support the findings of this study are available from the corresponding author upon reasonable request.

## Endnotes

^1^
“Cognitive control” can also be used to refer to “executive functions”. The two terms come from different research backgrounds but will indicate the same set of processes here.
